# Purification, Structural Characterization, and Anti-Inflammatory Effects of a Novel Polysaccharide Isolated from *Orostachys fimbriata*

**DOI:** 10.3390/molecules26237116

**Published:** 2021-11-24

**Authors:** Datong Hu, Fan Su, Gan Yang, Jing Wang, Yingying Zhang

**Affiliations:** 1 School of Pharmacy, Shandong University of Traditional Chinese Medicine, Jinan 250355, China; 17854111569@163.com (D.H.); sufan1123341185@163.com (F.S.); yg13808988390@163.com (G.Y.); 2 School of Traditional Chinese Medicine, Shandong University of Traditional Chinese Medicine, Jinan 250355, China

**Keywords:** *Orostachys fimbriata*, polysaccharide, α-(1,4)-glucan, anti-inflammatory activity

## Abstract

The present study elucidated the structural characteristics and anti-inflammatory activity of a novel polysaccharide isolated from *Orostachys fimbriata*, which is a traditional Chinese medicinal plant. *O. fimbriata* polysaccharide (OFP) was extracted and subsequently purified by chromatography using a DEAE cellulose-52 and Sephadex G-75 column. The molecular weight was determined as 6.2 kDa. HPGPC and monosaccharide composition analysis revealed a homogeneous polysaccharide containing only Glc. Chromatography and spectral analysis showed that the possible chemical structure consisted of →4)-α-Glcp-(1→ and a small quantity of →4,6)-β-Glcp-(1→ in the main chain and →6)-β-Glcp-(1→, α-Glcp-(1→, and β-Glcp-(1→ in the side chain. Morphological analysis using scanning electron microscopy (SEM) and atomic force microscopy (AFM) indicated that OFP had a multi-branched structure, and the sugar chain molecules of polysaccharide appeared aggregated. OFP was found to exhibit anti-inflammatory activity by reducing the secretion of inflammatory factors in RAW264.7 cells and by decreasing the extent of xylene-induced ear swelling in mice.

## 1. Introduction

The *Orostachys* genera, containing twelve species, is widely distributed in China, Korea, Mongolia, and Russia. *O. Japonicus* is the most studied plant of *Orostachys* spp., and it is well known for its immunomodulatory, anticancer, anti-inflammatory, and antioxidant activities [1,2,3,4]. In comparison to *O. japonicus*, *Orostachys fimbriata* has been used as a medicinal plant in China for more than 1000 years. The Chinese Pharmacopoeia (2020 version) records that extracts are effective in anti-inflammation and promoting wound healing. In addition, it is reported that *O. fimbriata*, combined with other herbs, exhibits neuroprotective activity in diabetic rats [5]. Previous studies on the chemical composition of *Orostachys* species revealed the presence of flavonoids, sugars, organic acid, sterols and triterpenoids [6,7,8]. However, until now, there are no published data on the chemical constituents of *O. fimbriata*.

Polysaccharides are important bioactive constituents of plants and animals [9]. These high-molecular weight polymers, consisting mainly of carbohydrates, are viewed as exceptional resources for the development of functional foods, biochemical, medicines, cosmetics, and biomaterials [10]. In particular, polysaccharides have attracted wide attention as medicines due to their broad spectrum of pharmacological functions including anti-oxidative, anti-microbial, anti-inflammatory, anticancer, and hypoglycemic activity [11,12,13,14,15]. Polysaccharides of differing structure show distinct biological functions [16]. Thus, elucidating the structure of polysaccharide will help further our understanding of their medicinal behavior.

Inflammation is part of the body’s immune response to infection or tissue damage. Normally, inflammation is beneficial for healing; however, it may be harmful if host tissues are compromised. Inflammation is regulated by pro-inflammatory molecules including interleukin-1β (IL-1β), interleukin-6 (IL-6), and tumor necrosis factor alpha (TNF-α), which are primarily produced by macrophages. Many polysaccharides are known to exhibit immunomodulatory effects, for example, a polysaccharide from *Umbilicaria yunnana* has been reported to reduce the release of inflammatory factors and exhibited a strong inhibitory effect on expression of IL-1β [17].

In this study, a novel polysaccharide, OFP, was isolated from *O. fimbriata* and analyzed in terms of molecular weight and monosaccharide composition. The structural characterization of OFP was elucidated using FT-IR spectroscopy, methylation analysis, and NMR spectroscopy. Morphological analysis was carried out using SEM and AFM. Furthermore, the anti-inflammatory activity of OFP was assessed in RAW264.7 cells and by measuring the extent of xylene-induced ear swelling in mice.

## 2. Results

### 2.1. Extraction and Purification of O. fimbriata Polysaccharide

**Figure 1:**
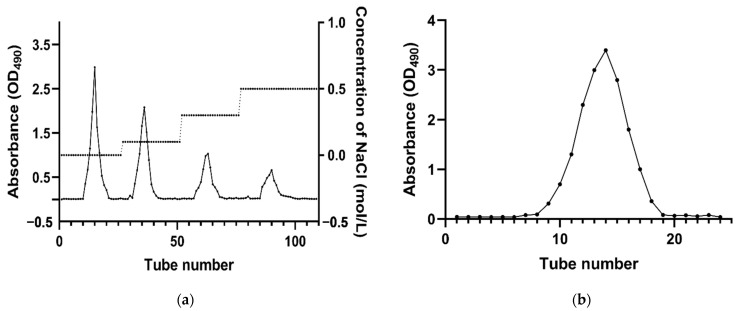
Elution curves of crude polysaccharides from *O. fimbriata* on DEAE cellulose-52 column (**a**) and Sephadex G-75 column (**b**).

The yield of crude polysaccharide from *O. fimbriata* was 5.7% (*w/w*) of the dry material. The crude polysaccharide was purified using a DEAE cellulose-52 column, giving rise to four distinct peaks in the elution profile (Figure 1a). Distilled water or NaCl solutions of varying concentration were employed as the eluent. Elution with distilled water resulted in the highest total carbon content and yield, and the resulting eluate was further purified using a Sephadex G-75 column. The elution curve is shown in Figure 1b. The main fraction (OFP) was collected and freeze-dried for determination of chemical composition and structure.

### 2.2. Homogeneity and Molecular Mass of OFP

**Figure 2:**
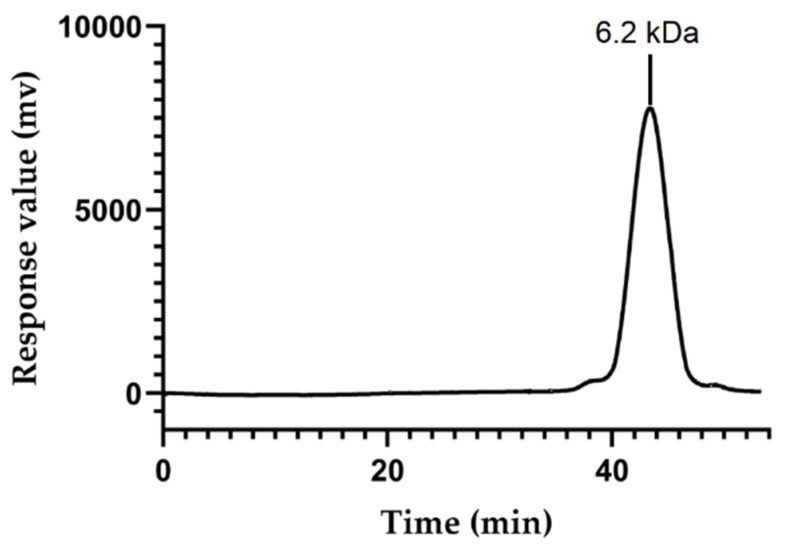
HPGPC profile of OFP.

The homogeneity and molecular weight of OFP were determined by HPGPC. The profile of OFP (Figure 2) appeared as a single, symmetrical peak with an absence of spurious peaks, indicating that OFP is a homogeneous polysaccharide. Calculations were based on a standard calibration curve of dextrans with different molecular weight (y = −0.1917x + 12.108, R^2^ = 0.9934), and the molecular weight (Mw) of OFP was estimated to be 6.2 kDa. It had been reported that the Mw of the polysaccharide extracted from *O. japonicus* (OJP) was 30–50 kDa [18]. The different molecular weight of OFP and OJP was not only due to different sources but also related to the extraction and purification methods of the polysaccharide. There were many studies showing that ultrasonic-assisted extraction would reduce the Mw of polysaccharides [19]. Hot-water extraction would cause polysaccharides particles to form large aggregates, but increasing the temperature, time, and ethanol concentration in the extraction leads to a decrease in the Mw of the polysaccharides obtained [20,21].

### 2.3. Total Carbohydrate Content and Monosaccharide Composition of OFP

**Figure 3:**
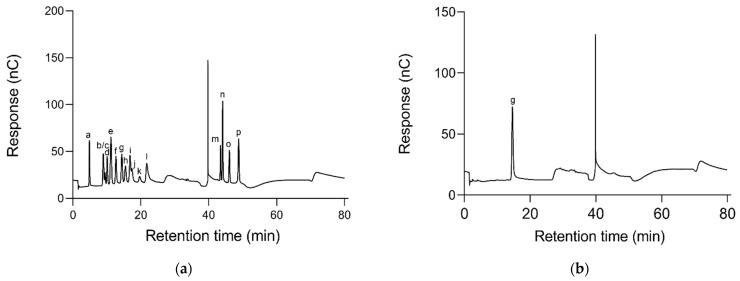
HPIC profiles of standard monosaccharides (**a**) and monosaccharide composition of OFP (**b**). a: Fuc b: GalN c: Rha d: Ara e: GlcN f: Gal g: Glc h: GlcNAc i: Xyl j: Man k: Fru l: Rib m: GalA n: GulA o: GlcA p: ManA.

The total sugar content of OFP was measured as 98.23%, and uronic acid and protein were not detected. The monosaccharide composition of OFP is shown in Figure 3b. OFP was found to be composed of Glc by comparing the retention time of standards (Figure 3a), confirming that OFP is a glucan-type polysaccharide [22].

### 2.4. UV and FT-IR Spectroscopy

**Figure 4:**
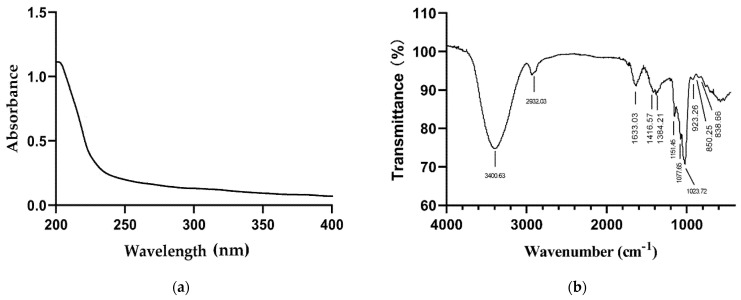
UV (**a**) and FT-IR (**b**) spectra of OFP.

The absence of peaks in the UV spectrum at 260 and 280 nm (Figure 4a) indicated that OFP was free of nucleic acid and proteins [23]. FT-IR spectroscopy at wavenumber within the range 4000–400 cm^−1^ was used for analysis of the structure and ring forms of OFP (Figure 4b). The spectrum featured the characteristic absorption peak of polysaccharide, the strong and wide bands around 3400.63 cm^−1^ due to -OH vibration, and a weak absorption band at 2932.03 cm^−1^, which is attributed to C-H stretching vibration [24]. The weak absorption bands located at 1633.03 cm^−1^ are ascribed to adsorbed water [25], while the absorption bands at 1416.57 cm^−1^ and 1384.21 cm^−1^ are assigned to C-H bending vibration [26]. Bands between 1000 and 1200 cm^−1^ are typical of C-O bending vibration [27]. The characteristic absorption band at 923.26 cm^−1^ suggested the presence of pyranoside configurations in OFP. The peaks at 850.25 cm^−1^ and 838.66 cm^−1^ were assigned to the presence of α-type and β-type glycosidic linkages [12,28].

### 2.5. Methylation Analysis

Methylation analysis is one of the principal means of exploring the primary structure of natural polysaccharides by way of monosaccharide residue content. The partially methylated alditol acetates (PMAAs) were determined to be 2,3,6-Me_3_-Glc, 2,3,4,6-Me_4_-Glc, 2,3,4–Me_3_-Glc, and 2,3-Me_2_-Glc, with the molar ratio of 3.11:1.78:1.50:1.00 (Table 1). OFP mainly contains four glycosidic linkage forms: 1,4-linked-Glcp, 1,6-linked-Glcp, T-linked-Glcp, and 1,4,6-linked-Glcp. 1,4-linked-Glcp accounts for the largest proportion, indicating that OFP was probably formed of 1,4-linked-glucans.

### 2.6. NMR Study

**Figure 5:**
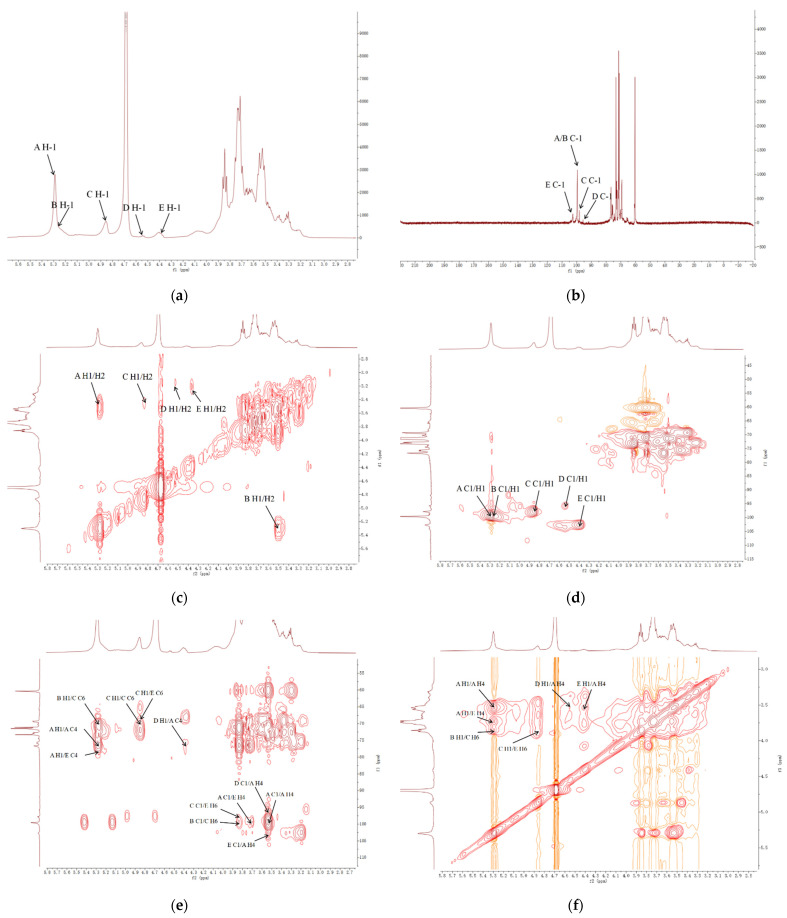

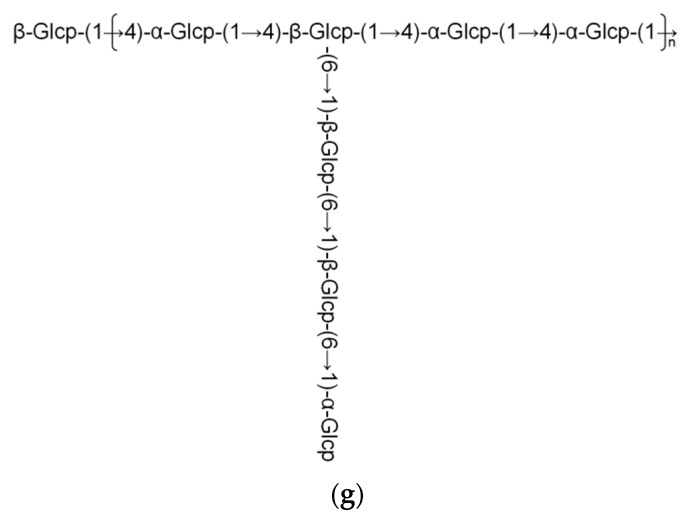
^1^H NMR (**a**), ^13^C NMR (**b**), ^1^H-^1^H COSY (**c**), HSQC (**d**), HMBC spectra (**e**), ^1^H-^1^H NOESY (**f**), and predicted structure of OFP (**g**).

OFP showed five anomeric proton signals at 5.29, 5.24, 4.84, 4.54, and 4.40 ppm respectively when probed by ^1^H spectrum (Figure 5a). Likewise, in the ^13^C spectrum (Figure 5b), five carbon signals were recorded at 102.66, 99.69, 99.55, 97.77, and 95.73 ppm. On examination of HSQC spectra (Figure 5d), signals of carbon and proton in the anomeric region were found at 5.29 (99.55), 5.24 (99.69), 4.84 (97.77), 4.54 (95.73), and 4.40 (102.66) ppm, which were distributed amongst residues A, B, C, D, and E, respectively. By analyzing the two-dimensional NMR spectrum, we obtained the signal values of C and H on the sugar ring, including the connection between sugar residues.

Residue A has a strong anomeric carbon and proton signal at 99.55 ppm (C-1) and 5.28 ppm (H-1), indicating →4)-α-Glcp-(1→. The chemical shifts from H-2 to H-5 were at 3.51, 3.82, 3.54, 3.84, and 3.73 ppm, corresponding to the cross-peaks in the ^1^H-^1^H COSY spectrum (Figure 5c). For C-2 to C-6, the corresponding ^13^C chemical shifts of carbon were detected at 71.35, 73.00, 76.55, 73.29, and 60.23 ppm, respectively. The existence of →4)-α-Glcp-(1→ was confirmed by the down-field chemical shift of C-4 and high-field chemical shift of C-6 [29]. Likewise, based on literature reports [23,30,31], residues B, C, D, and E were determined to be α-Glcp-(1→, →6)-β-Glcp-(1→, β-Glcp-(1→, and →6,4)-β-Glcp-(1→, respectively. The assignments of all the ^1^H NMR and ^13^C NMR chemical shifts are shown in Table 2.

By using HMBC spectrum, we obtained information about the remote H, C across the glycosidic bond. Thus, the linkage order and linkage position between sugar residues can be inferred. In the HMBC spectrum (Figure 5e), the cross-peak from A H-4 (δH 3.54) to A C-1 (δC 99.55) in the A residue confirmed that the inter-linkage of the main chain of →4)-α-D-Glcp-(1→ was at O-4. Peaks at 5.29/78.68 ppm, 99.55/3.72 ppm, and 102.66/3.54 ppm were assigned to A H-1/E C-4, A C-1/E H-4, and E C-1/A H-4, indicating linkages between C-4 of residue A and C-1 of residue E and between C-4 of residue E and C-1 of residue A. The cross peaks at 4.84/68.44 ppm and 97.77/3.82 ppm represented the connection between C-1 of residue C and C-6 of residue E. The signals at 5.24/70.16 ppm and 99.69/3.84 ppm were related to the inter-residue B H-1/C C-6 and B C-1/C H-6, further confirming that residues B and C were bridged as C-(6→1)-B. The cross-peaks at 4.84/70.16 ppm and 97.77/3.84 ppm were attributed to the inter-linkage of →6)-β-Glcp-(1→, implying that two residues C were linked at O-6. Linkage of residues D to C-4 of residues A was identified from examination of the cross-peak in the HMBC spectrum between D C-1 (δC 95.73) and A H-4 (δH 3.54). The cross-peaks at 5.29/3.54, 5.29/3.72, 4.40/3.54, 5.24/3.84, 4.84/3.84, and 4.54/3.54 ppm found in the NOESY spectra (Figure 5f) further confirmed the connection sequence of OFP and were ascribed to the inter-residue A H-1/A H-4, A H-1/E H-4, E H-1/A H-4, B H-1/C H-6, C H-1/C H-6, and D H-1/A H-4.

To summarize, combining the results of methylation analysis and NMR spectroscopy, we hypothesized that the main chain of OFP is likely composed of →4)-α-Glcp-(1→ and a small quantity of →4,6)-β-Glcp-(1→, while the side chain is probably formed from →6)-β-Glcp-(1→, α-Glcp-(1→ and β-Glcp-(1→. The proposed structure for OFP is presented in Figure 5g.

### 2.7. Morphological Characterization of OFP

**Figure 6:**
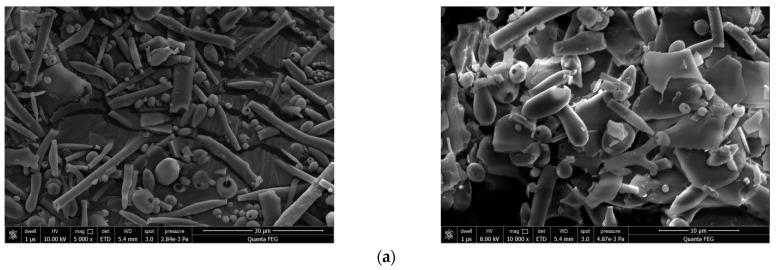

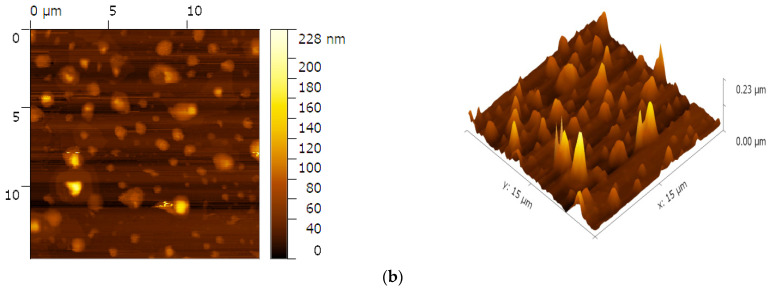
SEM (**a**) and AFM (**b**) image of OFP.

SEM is routinely used for visualization of the surface and internal structure and porosity of polysaccharides [32]. The morphology of OFP revealed by SEM (Figure 6a) consists mainly of sheet-like forms with a multitude of attached rod and spherical entities, suggesting that OFP forms a multiple-branching structure. Compared with the polysaccharide isolated from *Auricularia cornea*, OFP has a very similar structure, which may be caused by cavitation during ultrasonic-assisted extraction [33].

AFM enables high-resolution imaging of polysaccharide samples at the nanoscale, and it is routinely used to observe the conformation of individual macromolecules while obtaining quantitative information such as chain length and diameter [34]. The AFM image of OFP (Figure 6b) showed irregular polymer particles in water, suggesting that the sugar chain molecules of OFP were clustered together. The observed polymer particles were due to the formation of crosslinks between the polysaccharide molecules. In addition, the height of the OFP was 228 nm, which was much greater than the height of the single-chain polysaccharide molecule (0.1–1.0 nm), which also indicates that the molecular chains of OFP were intertwined to form a polymer.

### 2.8. Anti-Inflammatory Activity of OFP

#### 2.8.1. Effect of OFP on the Viability Cells and NO Production and Release of Inflammatory Factors of RAW264.7

**Figure 7:**
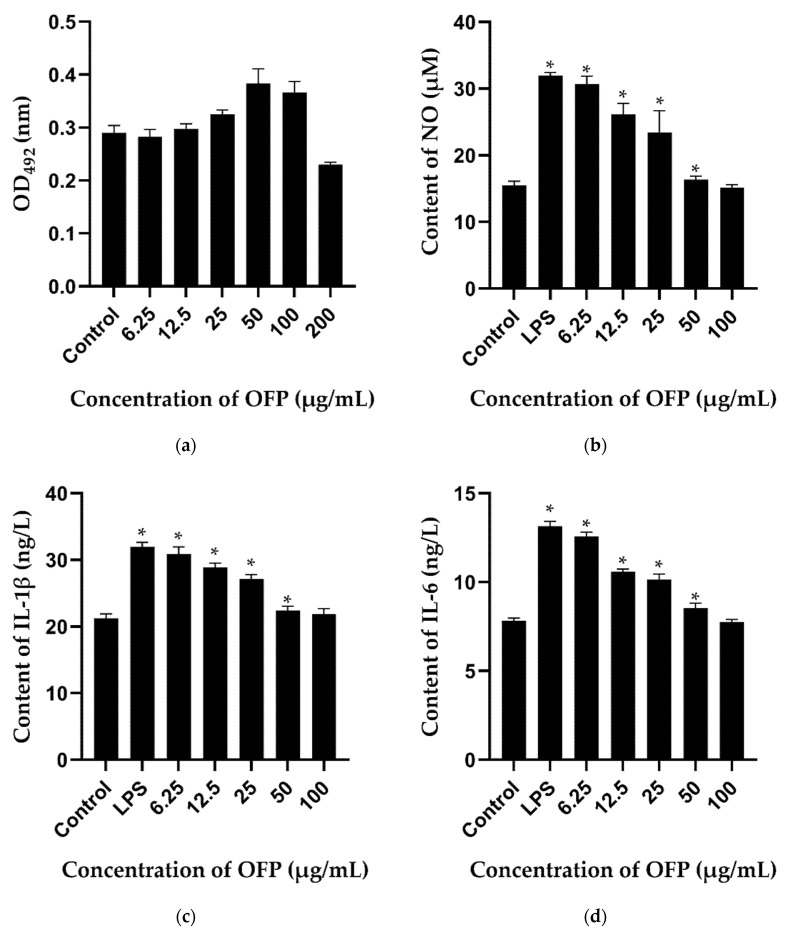
Effect of OFP on the viability of RAW264.7 cells and production of pro-inflammatory factors. Effect of OFP on (**a**) RAW264.7 cell proliferation, (**b**) NO production, (**c**) IL-1 β production, and (**d**) IL-6 production. * *p* < 0.05, as compared with the control group.

The cytotoxicity of OFP was assessed using the MTT method. As shown in Figure 7a, no effect was observed on the viability of RAW264.7 cells on exposure to OFP at concentrations of 6.25 to 100 μg/mL. Therefore, OFP was not toxic to RAW264.7 cells at all doses used in this study, which is consistent with the results for okra polysaccharides [13].

Macrophage is an important immune cell in the body, which has a variety of biological functions. LPS is one of the most abundant pathogen-associated molecular patterns (PAMPs) on the surface of Gram-negative bacteria, and pattern-recognition receptors (PRRs) are responsible for the recognition of PAMPs and activation of macrophages [35]. Activated macrophages exacerbate inflammation via the secretion of NO and inflammatory cytokines such as IL-1β and IL-6 [36]. LPS-induced inflammation models of RAW264.7 cells have been widely established to assess anti-inflammatory effects. The activated macrophages release a variety of inflammatory mediators, including NO, prostaglandins, and cytokines [37].

In this work, the anti-inflammatory effects of OFP on NO release and secretion of IL-1β and IL-6 were investigated. The results showed that OFP significantly inhibited LPS-mediated NO (Figure 7b), IL-1β (Figure 7c), and IL-6 (Figure 7d) production in RAW264.7 cells in a concentration-dependent manner. When the concentration of OFP was 100 μg/mL, the production of NO, IL-1β, and IL-6 was significantly reduced, and it is not statistically different with the control group, suggesting that OFP exerts its anti-inflammatory effect by reducing the production of NO and the secretion of inflammatory factors.

A number of polysaccharides have been shown to exhibit anti-inflammatory activity in vitro. For example, the marine red algae *Gracilaria caudata* polysaccharide was shown to possess an anti-inflammatory effect by reducing TNF-α and IL-1β levels [38]. Furthermore, *Ecklonia cava* polysaccharide was reported to inhibit LPS-induced iNOS and COX-2 gene expression as well as the subsequent production of NO and PGE2 by LPS-induced RAW264.7 macrophages in a concentration-dependent manner [39]. The immunomodulatory activity of polysaccharides is closely related to their structure, monosaccharide composition, and the type of glycosidic bond. The branched structure and enrichment of linkage may contribute to the biological activity of polysaccharides [40].

The extracts of *O. japonicus* also showed anti-inflammatory effects compared to *O. fimbriata*. The dichloromethane extract (DCM) of *O. japonicus* component significantly inhibited the mRNA levels of pro-inflammatory mediators and cytokines in LPS-stimulated cells. The anti-inflammatory effects of DCM have been reported to inhibit inflammatory responses through the inhibition of NF-κB activation and MAPK signaling, which is regulated by proteins upstream of MAPK and PI3K/Akt signaling pathways [3,4,41]. The 1,4,6-β-Glcp glycosidic bond in OFP might be an important mechanism contributing to the immunostimulatory activity.

#### 2.8.2. Effect of OFP on Ear Swelling in Mice

**Figure 8:**
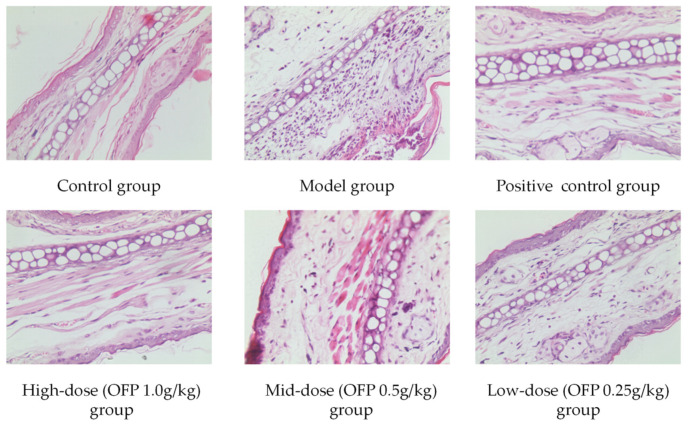
Effect OFP on ear-swelling response in mice. Tissue sections of mouse left auricle exposed to various concentrations of OFP. (H&E stain, 400X). Control group: No xylene applied; Model group: Application of xylene but no treatment; Positive control group: Application of xylene for treatment with aspirin; High/Mid/Low-dose group: Application of xylene for treatment with different concentrations of OFP.

Xylene-induced ear swelling in mice is regarded as a reliable model for evaluating anti-inflammatory behavior. Ear swelling is always accompanied by the release of pro-inflammatory factors, which result in increased capillary permeability and the inflammatory cell infiltration of surrounding tissues [42]. OFP inhibited xylene-induced ear swelling in mice by almost 50% at all concentrations tested (Table 3). H&E staining of tissue samples taken from the left auricle showed that OFP possessed anti-inflammatory activity at all concentrations tested, while doses of 1.0 g/kg and 0.5 g/kg significantly improved the lesion (Figure 8). In the untreated control group, which was not exposed to xylene, the subcutaneous connective tissue was closely arranged without edema and inflammatory cell infiltration. The ear tissue in the group exposed to xylene was significantly swollen and hypertrophic in comparison. In addition, skin ulcers were observed, and the subcutaneous connective tissue was arranged sparsely with inflammatory cell infiltration. The congestion, swelling, and inflammatory cell infiltration were alleviated in OFP groups compared with untreated animals, and the reduction was significant in the high/medium concentration OFP groups. The results show that OFP could reduce inflammation by inhibiting the release of inflammatory factors. Our findings demonstrated that OFP exhibits significant anti-oedematous and anti-inflammatory activity with potential for clinical application.

## 3. Materials and Methods

### 3.1. Materials and Reagents

Fresh *O. fimbriata* plants were collected from Yantai, Shandong Province, China. DMEM, fetal bovine serum (FBS), and PBS were obtained from Hyclone (Gibco, NY, USA). Sephadex G-75, lipopolysaccharide (LPS), Griess reagent, and different molecular weight dextrans (5, 11.6, 23.8, 48.6, 80.9, 148, 273, 409.8, and 667.8 kDa) were purchased from Sigma-Aldrich (St. Louis, MO, USA). DEAE-cellulose 52, dialysis bags, and monosaccharide standards (Fuc, GalN, Rha, Ara, GlcN, Gal, Glc, GlcNAc, Xyl, Man, Fru, Rib, GalA, GulA, GlcA, and ManA) were obtained from the Shanghai Yuanye Bio-Technology Co (China). Enzyme-linked immunosorbent assay (ELISA) kits for IL-1β and IL-6 were obtained from Meimian Biological Technology Co., Ltd. (Yancheng, Jiangsu, China). Other chemical reagents were of analytical grade and purchased from Sinopharm Chemical Reagent Co., Ltd. (Shanghai, China).

### 3.2. Isolation and Purification of Polysaccharides

*O. fimbriata* material was crushed and subsequently degreased in petroleum ether. The material was pre-extracted in anhydrous ethanol to remove small molecule impurities, such as pigment, lipids, and monosaccharides. After drying, the debris was extracted in distilled water under reflux at 80 °C for 2 h. The extraction was repeated twice, and the solutions obtained were combined and concentrated. Ethanol (95%) was added to achieve a final ethanol concentration of 80%, and the polysaccharide was precipitated. The precipitate was dissolved in water, deproteinated with Sevage reagent (chloroform: *n*-butanol = 4:1, *v/v*), repeated several times, and the supernatant was aspirated after centrifugation, filled into treated dialysis bags (cut-off: 3500 Da), dialyzed against distilled water, and freeze-dried to obtain *O. fimbriata* crude polysaccharide.

OFP was obtained by purification of the crude polysaccharide using DEAE cellulose-52 and Sephadex G-75 columns. The first stage involved the elution with 0.1 mol/L NaCl; 0.3 mol/L NaCl; and 0.5 mol/L NaCl solutions in distilled water using a DEAE cellulose-52 column (4.7 cm × 50 cm). The flow rate was controlled at 1.0 mL/min. The phenol–sulfuric acid method was used to monitor the eluted solutions (10 mL/tube) at 490 nm. The water-eluted solutions were collected, concentrated, dialyzed, and freeze-dried. After freeze-drying, the powder fraction was subjected to a second purification stage using a Sephadex G-75 column (1.7 cm × 80 cm) with ultrapure water at a flow rate of 0.5 mL/min and monitored at 490 nm using the phenol–sulfuric acid method. The final purified OFP was obtained after freeze drying of the eluate.

### 3.3. Physicochemical Characterization of OFP

The phenol sulfate method was used to determine the total sugar content of OFP [43], while the acidic sugar content was determined using the carbazole sulfate method [44]. The amount of protein was determined by the Coomassie brilliant blue G-250 assay [45].

The homogeneity and molecular mass of OFP was determined by HPGPC, using an Shimadzu LC-10A HPLC apparatus (Shimadzu, Kyoto, Japan) equipped with an BRT105-104-102 column (8 × 300 mm) (Biotech. Co., Ltd., Yangzhou, China).

### 3.4. Analysis of Monosaccharide Composition

The monosaccharide composition of OFP was detected by HPIC [46]. Briefly, OFP was hydrolyzed with 3 M TFA at 120 °C for 3 h. The resulting solution was accurately transferred to a centrifugal tube, dried by nitrogen, mixed with distilled water, and centrifuged to remove the supernatant. The monosaccharide composition was analyzed at 30 °C, using a Thermofisher ICS5000 system (Thermofisher, Waltham, MA, USA) equipped with Dionex carbopactmpa 20 column (3 μm × 150 nm) and an electrochemical detector at a flow rate of 0.3 mL/min. Fuc, GalN, Rha, Ara, GlcN, Gal, Glc, GlcNAc, Xyl, Man, Fru, Rib, GalA, GulA, GlcA, and ManA were used as standards.

### 3.5. UV and FT-IR Spectroscopy

OFP was prepared in deionized water to 1 mg/mL and scanned in the UV spectral range of 200 to 400 nm using UV-Vis spectrophotometer (Soptop UV9100B spectrophotometer, Ningbo, China). OFP was vacuum dried using P_2_O_5_ for 12 h; then, it was pressed with KBr and scanned in the wavenumber range of 4000 to 400 cm^−1^ using a Bruker tensor Ⅲ spectrometer (Bruker, Karlsruhe, Germany).

### 3.6. Methylation Analysis

Dried OFP was dissolved in 2 mL of anhydrous DMSO and ultrasonicated for 30 min. NaOH powder (20 mg) was added rapidly, and ultrasonication was continued for 20 min. The resulting solution was cooled and solidified in an ice water bath. Then, CH_3_I (15 mL) was added slowly, and the reaction solution was stirred intermittently for 3 h in the dark. The reaction was terminated by adding 2 mL of distilled water before adding 3 mL of trichloromethane for extraction. The lower organic phase was collected and extracted five times. TFA (2 mL, 2 M) was used to hydrolyze the dimethylated polysaccharide at 120 °C for 5 h. The hydrolyzed sample was reduced with NaBH_4_ and acetylated with acetic anhydride and pyridine. The partially methylated alditol acetates (PMAAs) were analyzed by GC-MS (Agilent 7890 7000D, CA, USA) equipped with an ion trap MS detector and EC-1 quartz capillary column (30 mm × 0.25 mm).

### 3.7. NMR Spectroscopy

OFP (50 mg) was dissolved in 99.9% D_2_O and freeze dried. The lyophilized sample was dissolved in 99.9% D_2_O and freeze dried again. The process was repeated three times. Signals of active hydrogen disappeared after D_2_O exchange. The 1D and 2D NMR spectra were recorded at 20 °C using a Bruker Advance Ш HD-600Mhz (Bruker, Karlsruhe, Germany).

### 3.8. Morphological Characterization of OFP

The macroscopic structure of OFP was studied using scanning electron microscopy (SEM) (FEI, Hillsboro, OR, USA). A small amount of OFP powder was fixed on a metal observation table, sprayed with gold powder, and then observed at different magnifications under an accelerating voltage of 8 or 10 keV.

The ultramicroscopic features of OFP were investigated using atomic force microscopy (AFM) (NSK Ltd., Tokyo, Japan). OFP was dissolved in distilled water and stirred magnetically at 50 °C for 2 h. The polysaccharide solution (pH = 6.8) was diluted to a concentration of 10 μg/mL, and 10 μL was dropped onto the surface of newly prepared mica sheets. Samples were dried in air overnight and analyzed in the tapping mode [22].

### 3.9. In Vitro Anti-Inflammatory Activity

#### 3.9.1. Cell Culture

RAW264.7 murine macrophage cells were obtained from the Beina Biology Cell bank (Beijing, China) and cultured in complete DMEM (containing 10% heat-inactivated FBS and 1% penicillin–streptomycin solution) at 37 °C in a 5% CO_2_ atmosphere.

#### 3.9.2. Assay of Cell Viability

The effect of OFP on the viability of RAW264.7 cells was investigated using the MTT method. RAW264.7 cells were incubated in 96-well plates at a concentration of 1 × 10^5^ cells/well in 100 μL of complete DMEM for 24 h. After this, cell monolayers were incubated in the presence or absence of OFP (100 μL) of different concentrations (0–200 μg/mL) for 24 h at 37 °C in 5% CO_2_. Next, MTT reagent (10 μL) was added, and the cells were incubated for 4 h. The medium was removed, and 150 μL DMSO were added to the wells to solubilize formazan crystals. ODs were measured at 492 nm in a microplate reader (Perlong, Beijing, China). Experiments were performed in triplicate.

#### 3.9.3. Determination of NO Production and Cytokine Assay

RAW264.7 cells (1 × 10^5^) were inoculated in 96-well plates and incubated for 24 h. Then, the cells were exposed to different concentrations (6.25–100 μg/mL) of OFP solution in combination with LPS (1 μg/mL). A blank control group (no LPS or OFP) and an LPS group (no OFP) were set up. After 24 h, cell supernatants were aspirated, and the NO concentration was measured using Griess reagent. The concentrations of IL-1β and IL-6 were measured by ELISA kits, according to the manufacturer’s instructions. The experiment was repeated three times.

### 3.10. In Vivo Anti-Inflammatory Activity

#### 3.10.1. Animal Experiments

The in vivo anti-inflammatory activity of OFP was evaluated in Balb/c mice (50% males, 50% females) purchased from Jinan Pengyue Experimental Animal Breeding Co., Ltd., Jinan, China (SCXK 2019 0003). Animals were fed and handled at 25 °C with a light/dark cycle of 12 h/12 h. The study complied with the regulations of the Animal Care and Use Committee of SDUTCM and was conducted in accordance with the Animal (Scientific Procedure) Law of 1986 (2013 version).

#### 3.10.2. In Vivo Anti-Inflammatory Activity

The anti-inflammatory activity of OFP was evaluated using the mouse ear-swelling assay [47]. Fifty BALB/c mice were randomly divided into five groups of 10 and fed by oral gavage with 50 mg/kg aspirin in saline solution (positive control), 2.0 mL/kg saline solution (model group), and 0.25, 0.5, and 1.0 g/kg OFP for seven consecutive days (low/mid/high-dose group). One hour after the last dose, 0.2 mL of xylene was applied uniformly to the inner and outer auricles of the right ear of the mice. After one hour, the thickness of the right ear of each animal was measured at the same location using vernier calipers, with the left ear as a control (control group). The swelling rate and swelling coefficient were calculated as previously reported [48]. Ear tissue was collected and stained with hematoxylin and eosin (H&E) for pathological observation.

### 3.11. Statistical Analysis

Data obtained from the study were presented as mean ± standard deviation (SD), and statistical analysis was performed using SPSS software (SPSS 17.0 for Windows; SPSS Inc, Chicago, IL, USA). One-way ANOVA was applied for comparison between multiple groups, and differences were considered statistically significant at the *p* < 0.05 level.

## 4. Conclusions

In this study, we firstly described the structure of polysaccharides extracted from *O. fimbriata*. The structure of the OFP was characterized by a molecular weight of 6.2 kDa and was composed of Glc. Structural analysis of OFP revealed that its main chain was composed of →4)-α-Glcp-(1→ and →4,6)-β-Glcp-(1→, and the branch contained →6)-β-Glcp-(1→, α-Glcp-(1→, and β-Glcp-(1→. Morphological characterization studies showed that OFP had a multi-branched structure. The polysaccharide molecular chains were intertwined and aggregated. In contrast to other *Orostachys* spp. such as *O. japonicus*, crude polysaccharides were extracted, but the structural analysis was not performed. OFP was shown to exhibit anti-inflammatory activity in vitro by inhibiting NO production and IL-1β and IL-6 release by LPS-activated macrophages. OFP also displayed significant anti-oedematous effects and anti-inflammatory activity in mice when tested using an ear-swelling assay. Compared with other polysaccharides, the immune activity of OFP may be closely related to its 1,4,6-β-Glcp bond and its complicated spatial structure. These results provide a theoretical basis for the application of OFP. In the future, in-depth research on the most important targets or pathways of the OFP will be performed to unveil the underlying mechanism. OFP has potential as an anti-inflammatory agent or functional food.

## Figures and Tables

**Table 1:** Methylation analysis data of OFP.

Methylated Alditol Acetates	Type of Linkage	Retention Time(min)	Molar Ratio	Mass Fragments (*m*/*z*)
2,3,6-Me_3_-Glc	1,4-Glcp	13.159	3.11	233, 187, 162, 142, 131, 118, 99, 87, 71
2,3,4,6-Me_4_-Glc	T-Glcp	11.548	1.78	205, 174, 162, 129, 118, 102, 87, 71
2,3,4-Me_3_-Glc	1,6-Glcp	13.362	1.50	233, 203, 189, 162, 143, 129, 102, 87, 71
2,3-Me_2_-Glc	1,4,6-Glcp	14.499	1.00	261, 201, 190, 159, 129, 118, 99, 89, 71

**Table 2:** Summary of ^1^H and ^13^C NMR chemical shifts of OFP.

Signals	Sugar Residues	C1	C2	C3	C4	C5	C6
		H1	H2	H3	H4	H5	H6
A	→4)-α-Glcp-(1→	99.55	71.35	73.00	76.55	73.29	60.23
		5.29	3.51	3.82	3.54	3.84	3.73
B	α-Glcp-(1→	99.69	74.42	70.87	75.60	72.65	61.18
		5.24	3.49	3.72	3.38	3.69	3.79
C	→6)-β-Glcp-(1→	97.77	74.42	71.77	70.16	74.47	70.16
		4.84	3.45	3.65	3.89	3.55	3.84
D	β-Glcp-(1→	95.73	70.59	71.47	73.19	73.29	62.60
		4.54	3.14	3.37	3.59	3.45	3.70
E	→6,4)-β-Glcp-(1→	102.66	73.00	72.53	78.68	71.87	68.44
		4.40	3.21	3.60	3.72	3.63	3.82

**Table 3:** Effect OFP on mice ear-swelling response.

Group	Drug Dose (g/L)	Ear Swelling (mg)	Swelling Inhibition Rate (%)
Control	-	12.27 ± 4.06	-
Positive control	0.05	5.95 ± 1.10 **	50.43
High-dose	10	6.07 ± 2.61 **	49.38
Medium-dose	5	7.94 ± 2.41 **	37.66
Low-dose	2.5	8.80 ± 2.24 **	29.72

** *p* < 0.01, as compared with the control.

## Data Availability

Data will be provided upon request.

## Sample Availability

Samples of the OFP are not available from the authors.

## References

[1] Lee H.Y., Park Y.M., Kim J., Oh H.G., Kim K.S., Kang H.J., Kim R.R., Kim M.J., Kim S.H., Yang H.J. (2019). Orostachys japonicus a. Berger Extracts Induce Immunity-Enhancing Effects on Cyclophosphamide-Treated Immunosuppressed Rats. BioMed Res. Int..

[2] Kim J.H., Nam G.S., Kim S.H., Ryu D.S., Lee D.S. (2019). Orostachys japonicus exerts antipancreatic cancer activity through induction of apoptosis and cell cycle arrest in PANC-1 cells. Food Sci. Nutr..

[3] Lee H.S. (2020). Orostachys japonicus extract inhibits the lipopolysaccharide-induced pro-inflammatory factors by suppression of transcription factors. Food Sci. Nutr..

[4] Lee H., Lee G., Kim S., Kim H., Suk D., Lee D. (2014). Anti-oxidizing effect of the dichloromethane and hexane fractions from Orostachys japonicus in LPS-stimulated RAW 264.7 cells via upregulation of Nrf2 expression and activation of MAPK signaling pathway. BMB Rep..

[5] Yu M.X., Lei B., Song X., Huang Y.M., Ma X.Q., Hao C.X., Yang W.H., Pan M.L. (2021). Compound XiongShao Capsule ameliorates streptozotocin-induced diabetic peripheral neuropathy in rats via inhibiting apoptosis, oxidative–nitrosative stress and advanced glycation end products. J. Ethnopharmacol..

[6] Kim J., Han S., Kwon J., Lee D. (2020). Orostachys japonicus ethyl acetate fraction suppresses MRSA biofilm formation. Asian Pac. J. Trop. Med..

[7] Jeong J.H., Ryu D.S., Suk D.H., Lee D.S. (2011). Anti-inflammatory effects of ethanol extract from Orostachys japonicus on modulation of signal pathways in LPS-stimulated RAW 264.7 cells. BMB Rep..

[8] Min-Jung K., Hwa-Hyun N., Myong-Soo C. (2020). Subcritical water extraction of bioactive compounds from Orostachys japonicus a. Berger (Crassulaceae). Sci. Rep.UK.

[9] Yu Y., Shen M., Song Q., Xie J. (2018). Biological activities and pharmaceutical applications of polysaccharide from natural resources: A review. Carbohydr Polym.

[10] Baldwin A.D., Kiick K.L. (2010). Polysaccharide-modified synthetic polymeric biomaterials. Biopolymers.

[11] Cheng H., Huang G. (2018). Extraction, characterization and antioxidant activity of Allium sativum polysaccharide. Int. J. Biol. Macromol..

[12] Kungel P., Correa V.G., Correa R., Peralta R.A., Sokovic M., Calhelha R.C., Bracht A., Ferreira I., Peralta R.M. (2018). Antioxidant and antimicrobial activities of a purified polysaccharide from yerba mate (*Ilex paraguariensis*). Int. J. Biol. Macromol..

[13] Liu Y., Ye Y., Hu X., Wang J. (2021). Structural characterization and anti-inflammatory activity of a polysaccharide from the lignified okra. Carbohyd. Polym..

[14] Sahayanathan G.J., Padmanaban D., Raja K., Chinnasamy A. (2020). Anticancer effect of purified polysaccharide from marine clam Donax variabilis on A549 cells. J. Food Biochem..

[15] Cao C., Li C., Chen Q., Huang Q., Perez M., Fu X. (2019). Physicochemical characterization, potential antioxidant and hypoglycemic activity of polysaccharide from Sargassum pallidum. Int. J. Biol. Macromol..

[16] Gao X., Qu H., Shan S., Song C., Baranenko D., Li Y., Lu W. (2020). A novel polysaccharide isolated from Ulva Pertusa: Structure and physicochemical property. Carbohyd. Polym..

[17] Luo D., Wang Z., Zhou R., Cao S. (2020). A polysaccharide from Umbilicaria yunnana: Structural characterization and anti-inflammation effects. Int. J. Biol. Macromol..

[18] Ryu D., Baek G., Kim E., Kim K., Lee D. (2010). Effects of polysaccharides derived from Orostachys japonicus on induction of cell cycle arrest and apoptotic cell death in human colon cancer cells. BMB Rep..

[19] Du B., Zeng H., Yang Y., Bian Z., Xu B. (2016). Anti-inflammatory activity of polysaccharide from Schizophyllum commune as affected by ultrasonication. Int. J. Biol. Macromol..

[20] Chen G., Fang C., Ran C., Tan Y., Yu Q., Kan J. (2019). Comparison of different extraction methods for polysaccharides from bamboo shoots (*Chimonobambusa quadrangularis*) processing by-products. Int. J. Biol. Macromol..

[21] Yuan Y., Zou P., Zhou J., Geng Y., Fan J., Clark J., Li Y., Zhang C. (2019). Microwave-assisted hydrothermal extraction of non-structural carbohydrates and hemicelluloses from tobacco biomass. Carbohyd. Polym..

[22] Gong Y., Cao C., Ai C., Wen C., Wang L., Zhao J., Han Y., Song S., Xiao H. (2020). Structural characterization and immunostimulatory activity of a glucan from Cyclina sinensis. Int. J. Biol. Macromol..

[23] Yuan Q., Zhang J., Xiao C., Harqin C., Ma M., Long T., Li Z., Yang Y., Liu J., Zhao L. (2020). Structural characterization of a low-molecular-weight polysaccharide from Angelica pubescens Maxim. F. Biserrata Shan et Yuan root and evaluation of its antioxidant activity. Carbohyd. Polym..

[24] Ye G., Li J., Zhang J., Liu H., Ye Q., Wang Z. (2021). Structural characterization and antitumor activity of a polysaccharide from Dendrobium wardianum. Carbohyd. Polym..

[25] Wu Q., Luo M., Yao X., Yu L. (2020). Purification, structural characterization, and antioxidant activity of the COP-W1 polysaccharide from *Codonopsis tangshen* Oliv. Carbohyd. Polym..

[26] Vasilieva T., Sigarev A., Kosyakov D., Ul’Yanovskii N., Anikeenko E., Chuhchin D., Ladesov A., Hein A.M., Miasnikov V. (2017). Formation of low molecular weight oligomers from chitin and chitosan stimulated by plasma-assisted processes. Carbohydr. Polym..

[27] Li G., Chen P., Zhao Y., Zeng Q., Ou S., Zhang Y., Wang P., Chen N., Ou J. (2021). Isolation, structural characterization and anti-oxidant activity of a novel polysaccharide from garlic bolt. Carbohyd. Polym..

[28] Cheng Y., Xie Y., Ge J., Wang L., Peng D., Yu N., Zhang Y., Jiang Y., Luo J., Chen W. (2021). Structural characterization and hepatoprotective activity of a galactoglucan from Poria cocos. Carbohyd. Polym..

[29] Xiong Q., Luo G., Zheng F., Wu K., Yang H., Chen L., Tian W. (2021). Structural characterization and evaluation the elicitors activity of polysaccharides from Chrysanthemum indicum. Carbohyd. Polym..

[30] Li J., Gu F., Cai C., Hu M., Fan L., Hao J., Yu G. (2020). Purification, structural characterization, and immunomodulatory activity of the polysaccharides from Ganoderma lucidum. Int. J. Biol. Macromol..

[31] Huo J., Lei M., Zhou Y., Zhong X., Liu Y., Hou J., Long H., Zhang Z., Tian M., Xie C. (2021). Structural characterization of two novel polysaccharides from *Gastrodia elata* and their effects on *Akkermansia muciniphila*. Int. J. Biol. Macromol..

[32] Ktari N., Bkhairia I., Nasri M., Ben S.R. (2020). Structure and biological activities of polysaccharide purified from Senegrain seed. Int. J. Biol. Macromol..

[33] Wang Y., Guo M. (2020). Purification and structural characterization of polysaccharides isolated from Auricularia cornea var. Li. Carbohydr. Polym..

[34] de Oliveira S.R.F., de Franca D.F., Silva M., Brito L.M., Pessoa C., de Lima L., de Paula R., de Souza D.A.L.J., de Araujo A.R., Da S.D. (2020). Anti-proliferative profile of Anacardium occidentale polysaccharide and characterization by AFM. Int. J. Biol. Macromol..

[35] Mazgaeen L., Gurung P. (2020). Recent advances in lipopolysaccharide recognition systems. Int. J. Mol. Sci..

[36] Shapiro H., Lutaty A., Ariel A. (2011). Macrophages, meta-inflammation, and immuno-metabolism. Sci. World J..

[37] Zhu T., Zhang W., Feng S.J., Yu H.P. (2016). Emodin suppresses LPS-induced inflammation in RAW264.7 cells through a PPARgamma-dependent pathway. Int. Immunopharmacol..

[38] Chaves L.S., Nicolau L.A., Silva R.O., Barros F.C., Freitas A.L., Aragao K.S., Ribeiro R.A., Souza M.H., Barbosa A.L., Medeiros J.V. (2013). Antiinflammatory and antinociceptive effects in mice of a sulfated polysaccharide fraction extracted from the marine red algae *Gracilaria caudata*. Immunopharmacol. Immunotoxicol..

[39] Lee W.W., Ahn G., Arachchillage J.P., Kim Y.M., Kim S.K., Lee B.J., Jeon Y.J. (2011). A polysaccharide isolated from Ecklonia cava fermented by Lactobacillus brevis inhibits the inflammatory response by suppressing the activation of nuclear factor-kappaB in lipopolysaccharide-induced RAW 264.7 macrophages. J. Med. Food.

[40] Dong Z., Zhang M., Li H., Zhan Q., Lai F., Wu H. (2020). Structural characterization and immunomodulatory activity of a novel polysaccharide from *Pueraria lobata* (Willd.) Ohwi root. Int. J. Biol. Macromol..

[41] Lee H., Ryu D., Lee G., Lee D. (2012). Anti-inflammatory effects of dichloromethane fraction from Orostachys japonicus in RAW 264.7 cells: Suppression of NF-κB activation and MAPK signaling. J. Ethnopharmacol..

[42] Richardson J.D., Vasko M.R. (2002). Cellular mechanisms of neurogenic inflammation. J. Pharmacol. Exp. Ther..

[43] Chen S.J., Li J.Y., Zhang J.M. (2019). Extraction of yellow pear residue polysaccharides and effects on immune function and antioxidant activity of immunosuppressed mice. Int. J. Biol. Macromol..

[44] An Q., Ye X., Han Y., Zhao M., Chen S., Liu X., Li X., Zhao Z., Zhang Y., Ouyang K. (2020). Structure analysis of polysaccharides purified from Cyclocarya paliurus with DEAE-Cellulose and its antioxidant activity in RAW264.7 cells. Int. J. Biol. Macromol..

[45] Rjeibi I., Feriani A., Hentati F., Hfaiedh N., Michaud P., Pierre G. (2019). Structural characterization of water-soluble polysaccharides from Nitraria retusa fruits and their antioxidant and hypolipidemic activities. Int. J. Biol. Macromol..

[46] Liu S., Yang Y., Qu Y., Guo X., Yang X., Cui X., Wang C. (2020). Structural characterization of a novel polysaccharide from Panax notoginseng residue and its immunomodulatory activity on bone marrow dendritic cells. Int. J. Biol. Macromol..

[47] Gad S.C., Dunn B.J., Dobbs D.W., Reilly C., Walsh R.D. (1986). Development and validation of an alternative dermal sensitization test: The mouse ear swelling test (MEST). Toxicol. Appl. Pharmacol..

[48] Jouini M., Abdelhamid A., Chaouch M.A., le Cerf D., Bouraoui A., Majdoub H., Ben J.H. (2018). Physico-chemical characterization and pharmacological activities of polysaccharides from *Opuntia microdasys* var. Rufida cladodes. Int. J. Biol. Macromol..

